# A Cuproptosis-Related lncRNAs Signature Could Accurately Predict Prognosis in Patients with Clear Cell Renal Cell Carcinoma

**DOI:** 10.1155/2022/4673514

**Published:** 2022-12-22

**Authors:** Wei Zhang, Han Wang, Wei Wang, Haoqiang Xue, Maolin Qiao, Liying Song, Shuang Wang, Zhaoyu Ren, Zhifang Ma

**Affiliations:** ^1^Department of Urology, First Hospital of Shanxi Medical University, Taiyuan, Shanxi, China; ^2^Department of Clinical Medicine, Shanxi Medical University, Taiyuan, Shanxi, China; ^3^Department of Urology, Second Hospital of Shanxi Medical University, Taiyuan, Shanxi, China

## Abstract

**Background:**

Clear cell renal cell carcinoma (ccRCC) is the most common subtype of kidney cancers. As cuproptosis, a new cell death mechanism proposed recently, differs from all other known mechanisms regulating cell death, we aimed to create prognostic markers using cuproptosis-related long non-coding ribonucleic acids (RNAs; lncRNAs) and elucidate the molecular mechanism.

**Methods:**

Data from transcriptome RNA sequencing of ccRCC samples and the relevant clinical data were downloaded from The Cancer Genome Atlas, and Pearson's correlation analysis was implemented to obtain the cuproptosis-related lncRNAs. Then, univariate Cox, multivariate Cox, and Least Absolute Shrinkage and Selection Operator Cox analyses were performed to construct the risk signatures. The cuproptosis-related lncRNAs predictive signature was evaluated with receiver operating characteristic curves and subgroup analysis. Finally, Gene Set Enrichment Analysis (GSEA), single-sample GSEA (ssGSEA), tumor immune microenvironment (TIME), and immune checkpoints were performed to explore the relationship between immunity and patient prognosis.

**Results:**

Five cuproptosis-related lncRNAs, including FOXD2-AS1, LINC00460, AC091212.1, AC007365.1, and AC026401.3, were used to construct the signature. In the training and test sets, low-risk groups (as identified by a risk score lower than the median) demonstrated a better prognosis with an area under the curve for 1-, 3-, and 5-year survival being 0.793, 0.716, and 0.719, respectively. GSEA analysis suggested significant enrichment of the tricarboxylic acid cycle and metabolism-related pathways in the low-risk group. Besides, both ssGSEA and TIME suggested that the high-risk group exhibited more active immune infiltration.

**Conclusion:**

We proposed a cuproptosis-related lncRNAs signature, which had the potential for prognoses and prediction. Our findings might contribute to elucidating potential genomic biomarkers and targets for future therapies in the cuproptosis-related signaling pathways.

## 1. Introduction

The most frequent parenchymal lesion of the kidney is renal cell carcinoma (RCC), which takes up about 90% of all renal malignancies [1] and 3% of all cancers [2]. According to European Association of Urology (EAU) guidelines, RCC incidence rises on an annual basis with the annual growth rate being about 2% [2]. Particularly, clear cell renal cell carcinoma (ccRCC) is responsible for around 75% of all RCC cases and more than 175,000 deaths each year in the world, thereby being the most commonly seen histological subtype of RCC [2, 3]. Distant metastases have been reported in 30–35% of surgical patients [3]. Cancer prognoses and survival prediction, on the other hand, are important to both doctors and patients. Most current prognostic analyses of RCC are primarily based on clinical data, ignoring changes in the microenvironment with the progression of ccRCC. The microenvironment frequently changes during tumor development, resulting in significant expression differences of the specific genes [4]. As a result, developing prognostic prediction models of the microenvironment is in demand to identify potential biomarkers and therapeutic targets.

Cuproptosis, a new cell death mechanism proposed recently, differs from all the other recognized mechanisms of programmed cell death [5]. A previous study has demonstrated that it is the protein lipidation that mediates copper-induced cell death, which is a highly conserved post-translational modification of lysine known to occur in merely four cell death-related enzymes. Although the tricarboxylic acid (TCA) cycle is inhibited, excess copper can promote the aggregation of lipidated proteins, thereby disrupting the TCA cycle, destabilizing Fe–S cluster proteins, inducing proteotoxic stress, and eventually leading to cell death [5]. However, whether copper-mediated cell death is involved in ccRCC remains unknown.

Long non-coding ribonucleic acids (RNAs; lncRNAs) are RNA chains longer than 200 base pairs without providing protein-coding capacities. Even so, these RNAs participate in regulating target gene expression as well as many cellular processes, linked to the occurrence of cancer [6–8]. Many studies have shown that lncRNAs, such as lnc-ROR [9], lnc-ITGB1 [10], and Lnc-DYNC2H1-4 [11], are involved in regulating tumor metastasis. These findings suggest that regulating lncRNAs may become a new therapeutic approach for human cancers. However, no studies have focused on a systemic evaluation of cuproptosis-related lncRNAs signatures as well as their association with the overall survival (OS) of ccRCC patients. Therefore, this study addresses such a problem by first creating prognostic markers for multiple differentially expressed lncRNAs related to cuproptosis in ccRCC with The Cancer Genome Atlas (TCGA) data.

## 2. Method

### 2.1. Data Acquisition and Preprocessing

The Fragments Per Kilobase Million data of transcriptome RNA sequencing of samples from 611 ccRCC patients and the relevant clinical data were acquired from TCGA (https://portal.gdc.cancer.gov/). To minimize statistical bias, patients who lacked OS data or revealed poor OS (<30 days) were ruled out, and data of 513 ccRCC patients were included in the subsequent analysis. 10 cuproptosis-related genes were adopted from recent studies [5]. The 513 ccRCC patients were randomized into one test set and one training set at a ratio of 1 : 1 by the caret R package.

### 2.2. Data Processing of lncRNAs and Cuproptosis-Related Genes

The correlations between candidate lncRNAs and cuproptosis-related genes were calculated via the Pearson's correlation analysis. Cuproptosis-related lncRNAs were defined as having correlation coefficient (|*R*|) > 0.4 and *p* < 0.05. Following that, lncRNAs with differential expressions between the tumor group and the paracancer group were identified with a threshold of log2 fold change (logFC) > 1 and false discovery rate < 0.05 using the limma R package in the R software V-4.1.0 (https://www.rproject.org/).

### 2.3. The Cuproptosis-Related lncRNAs Predictive Signature

The training set was implemented to establish a cuproptosis-related lncRNAs signature, which was validated with the test set and all available data. First, univariate Cox (uni-Cox) regression analysis revealed 62 cuproptosis-related lncRNAs that are significantly correlated with ccRCC prognosis (*p* < 0.05). The glmnet R package was applied to perform the Least Absolute Shrinkage and Select Operator (LASSO) Cox analysis with an estimated penalty parameter of 10-fold cross-validation to prevent over-fitting. From the LASSO Cox analysis, 9 optimal lncRNAs associated with ccRCC prognosis were identified. Finally, multivariate Cox (multi-Cox) regression analysis was implemented to get the cuproptosis-related lncRNAs for the construction of the predictive signature, and the calculation formula for risk scores was:

(1) $$\begin{array}{c} \text{Risk score}=\overset{n}{\underset{i=1}{\sum }}{\text{EXP}}_{i}\times {\text{coefficient}}_{i}. \end{array}$$

The coefficients EXP*_i_* and coefficient*_i_* represented each lncRNA expression level and the regression coefficients of the multi-Cox regression analysis for each lncRNA, respectively. With the constructed predictive signature, each patient was given a risk score, and patients were assigned into either the high-risk or low-risk group based on the comparison results between their risk scores and the median score.

### 2.4. Evaluation of the Cuproptosis-Related lncRNAs Predictive Signature

The Kaplan–Meier (KM) method was implemented to assess the survival of the two risk groups, and log-rank statistical methods were applied to compare the survival data. The patients were separated into subgroups to analyze model stability by clinicopathological factors. The forest maps were used to illustrate the findings of uni-Cox regression and multi-Cox analyses to see if the risk score is an independent indicator of ccRCC prognosis. The receiver operating characteristic curves were depicted to evaluate the model accuracy. Besides, a nomogram was created with the clinicopathological parameters (e.g., age, gender, disease grade, and disease stage) and the risk scores to predict the 1-, 3-, and 5-year survival of ccRCC patients. A calibration curve was used to see if the anticipated survival rate fitted the authentic one.

### 2.5. Estimation of the Tumor Immune Microenvironment with the Prognostic Signature

The prognostic differences between different groups were studied with the Gene Set Enrichment Analysis (GSEA) software V4.2.1. Following that, using the GSVA R package, ccRCC-infiltrating immune cells and immunological function were scored using single-sample GSEA (ssGSEA). Multi-boxplots are used to display the scores of the two groups in terms of their immune cells and functions. To find out if there is a correlation between the established signature and tumor immune microenvironment (TIME), seven techniques were implemented to generate immune cells infiltration data for TCGA-KIRC dataset samples, namely XCELL, MCPCOUNTER, QUANTISEQ, EPIC, CIBERSORT, TIMER, and CIBERSORT-ABS [12–18]. The association between the immune cell subpopulations and the risk score was investigated with the Spearman's correlation analysis. The quantity of stromal and immune cells in different groups was then investigated using the estimate R package. Each patient's StromalScore, ImmuneScore, and ESTIMATE Score (StromalScore + ImmuneScore) were determined. To estimate the response of high- and low-risk groups to immune-checkpoint blockade (ICB), Immune Cell Abundance Identifier (ImmuCellAI) (http://bioinfo.life.hust.edu.cn/ImmuCellAI/) was used. The Wilcoxon signed-rank test was implemented to evaluate the differences between these scores, and a *p* < 0.05 was considered significant. Finally, the Wilcoxon signed-rank test (the limma R package) was implemented to compare the expression levels of the immune checkpoints between the two risk groups.

### 2.6. Statistical Analysis

The R 4.1.0 was used for statistical analysis. The Wilcoxon test was implemented to find the differences in expression of cuproptosis-related lncRNAs between tumor and normal. The correlations between candidate lncRNAs and cuproptosis-related genes were calculated via the Pearson's correlation analysis. The KM method was implemented to assess the survival of the different risk groups. The uni-Cox regression and multi-Cox regression analyses were used to see if the risk score is an independent indicator of ccRCC prognosis. The association between the immune cell subpopulations and the risk score was investigated with the Spearman's correlation analysis. The Wilcoxon test was implemented to evaluate the differences between two group in tumor immune microenvironment and immune checkpoints aspects. The *p* < 0.05 was considered statistically significant.

## 3. Results

### 3.1. Cuproptosis-Related lncRNAs in ccRCC Patients

**Figure 1:**
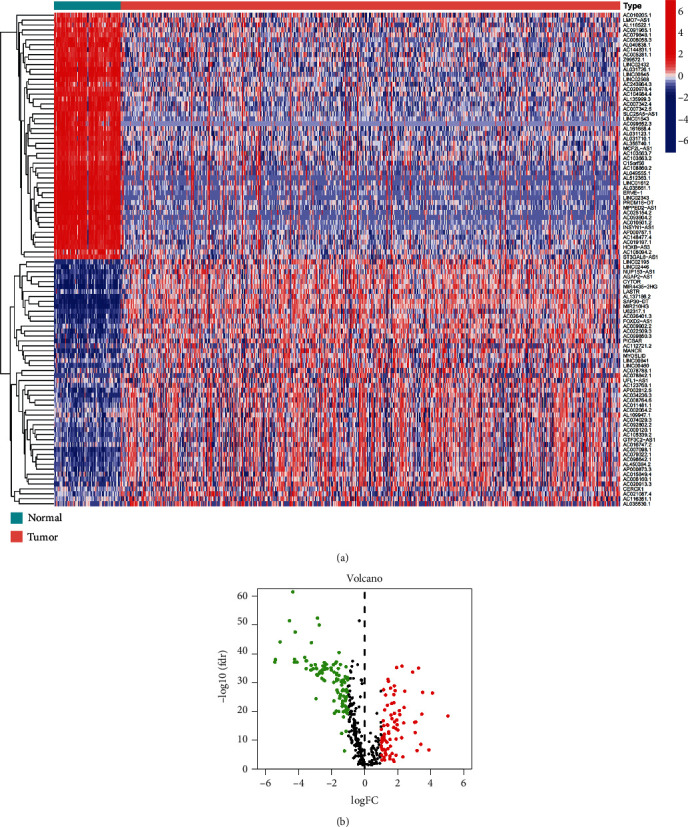
Identification of cuproptosis-related differential long non-coding ribonucleic acids (lncRNAs) in clear cell renal cell carcinoma. (a) Heatmap of 50 most significantly regulated cuproptosis-related lncRNAs between tumor and paracancer samples, respectively. (b) A volcano map of differential cuproptosis-related lncRNAs.

We found 14,056 lncRNAs in the ccRCC transcriptome data retrieved from TCGA using GTF files. Through co-expression analysis, 417 cuproptosis-related lncRNAs were obtained based on the 10 cuproptosis-related genes. A total of 184 cuproptosis-related differential lncRNAs, comprising 103 downregulated lncRNAs and 81 upregulated lncRNAs, were discovered in tumor and paracancer samples (Figures 1(a) and 1(b)). The clinical information of 513 ccRCC patients was displayed in Table 1. The diagram of our study flow is provided in Supplementary Figure S1.

### 3.2. The Cuproptosis-Related lncRNAs Predictive Signature Was Created

**Figure 2:**
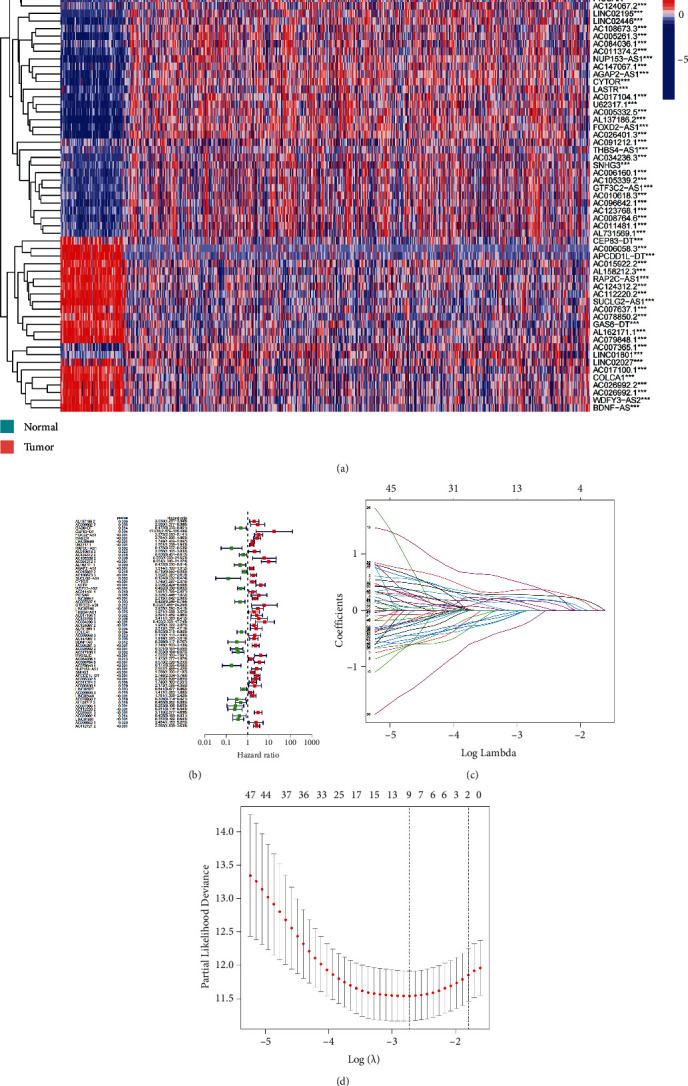
The prognostic model for cuproptosis-related long non-coding ribonucleic acids (lncRNA) signatures. (a) and (b) The heatmap and forest plot of prognosis-related lncRNAs as obtained from uni-Cox analysis. (c) and (d) The Least Absolute Shrinkage and Select Operator regression results for further lncRNA screening.

We found 62 cuproptosis-related lncRNAs that were substantially linked with OS in the train set using uni-Cox regression analysis and created a forest map and heat map using them (Figures 2(a) and 2(b)). We used LASSO regression analysis and identified 9 cuproptosis-related lncRNAs in ccRCC with the first-rank value of log(*λ*) being the least likelihood of deviation (Figures 2(c) and 2(d)). Following that, we used multi-Cox regression analysis to create a prediction signature consisting of 5 cuproptosis-related lncRNAs (FOXD2-AS1, LINC00460, AC091212.1, AC007365.1, and AC026401.3; Supplementary Table S1). Following that, each ccRCC patient had their risk score determined with the correlation coefficients from multi-Cox regression analysis, and they were assigned into one of the two risk groups based on their risk scores.

### 3.3. Cuproptosis-Related lncRNAs Signature Prognosis Values

**Figure 3:**
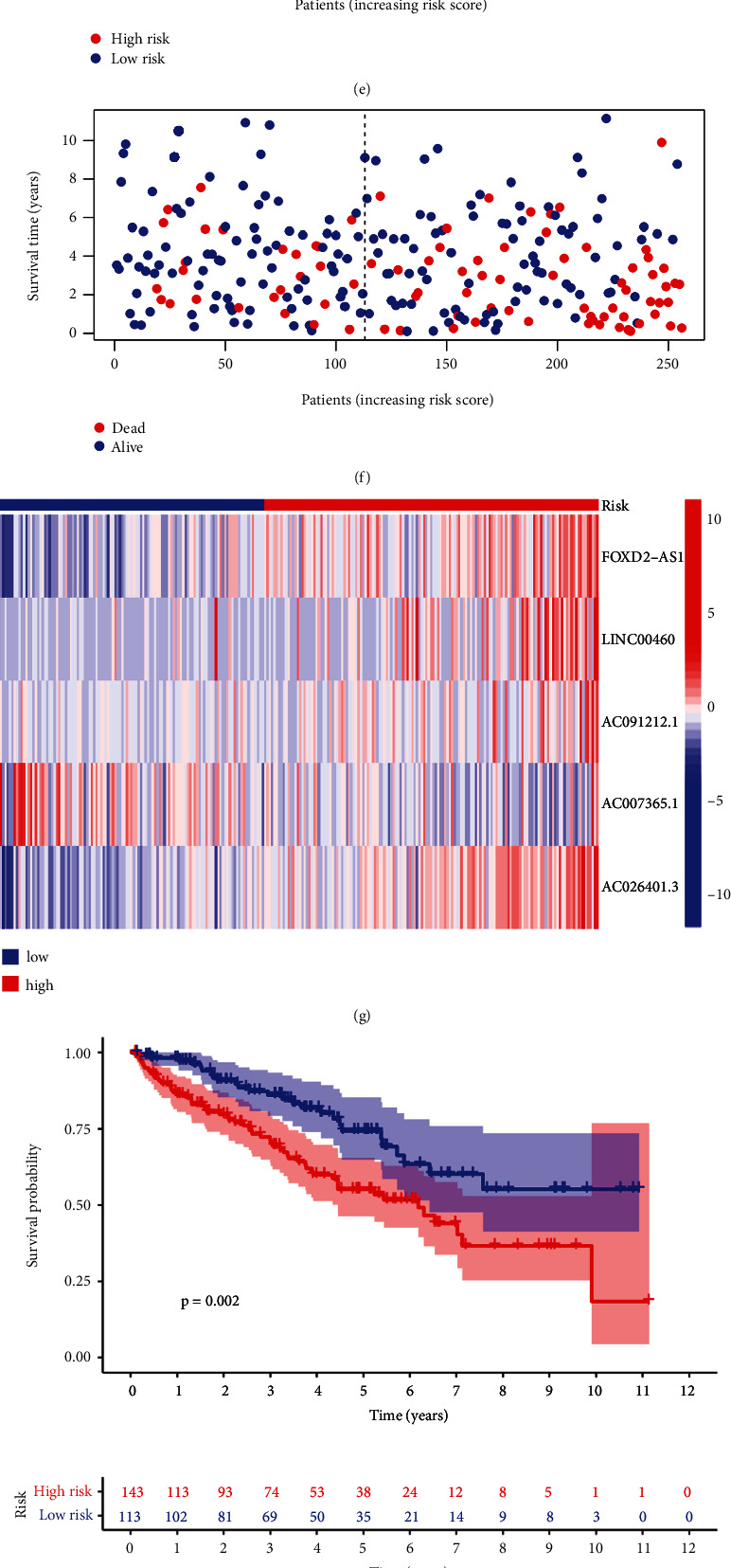
Prognostic values of the 5 cuproptosis-related long non-coding ribonucleic acids (lncRNAs) signatures. (a) Scatter plot of the distribution of risk scores from low to high in the training set. (b) Scatter plot of different risk scores corresponding to the survival statuses in the training set. (c) Heatmap of lncRNAs expression in the training set patients with different risk scores. (d) Survival curves of the training set patients. (e), (f), (g), and (h) Corresponding plots in the testing group. (i), (j), (k), and (l) Corresponding plots in the whole group.

**Figure 4:**
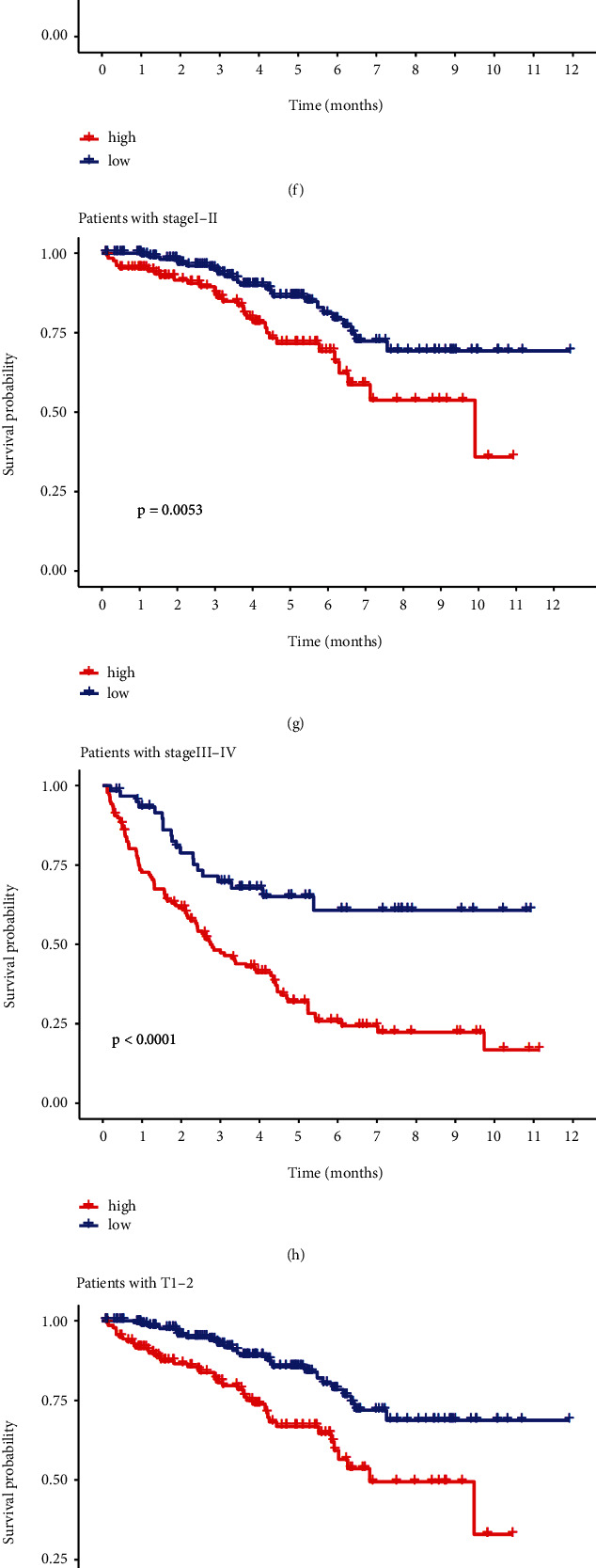
Subgroup analysis of risk characteristics. Subgroup analyses split by (a) and (b) age; (c) and (d) gender; (e) and (f) pathological grade; (g) and (h) stage; (i) and (j) tumor stage; (k) and (l) node stage; and (m) and (n) metastasis stage.

Five cuproptosis-related lncRNAs were evaluated for their risk score distributions, survival times, survival status, and relevant expression in the two risk groups in the training and test sets as well as the overall data set to assess the risk signature's predictive capacity (Figures 3(a), 3(b), 3(c), 3(d), 3(e), 3(f), 3(g), 3(h), and 3(l)). Consistent with the former subgroup analyses by clinicopathological factors, the results suggested a better prognosis in the low-risk group apart from N1 and M1, confirming the stability of our signature (Figures 4(a), 4(b), 4(c), 4(d), 4(e), 4(f), 4(g), 4(h), and 4(l)).

### 3.4. The Cuproptosis-Related lncRNAs Signature Is an Independent Indicator for ccRCC Prognosis

**Figure 5:**
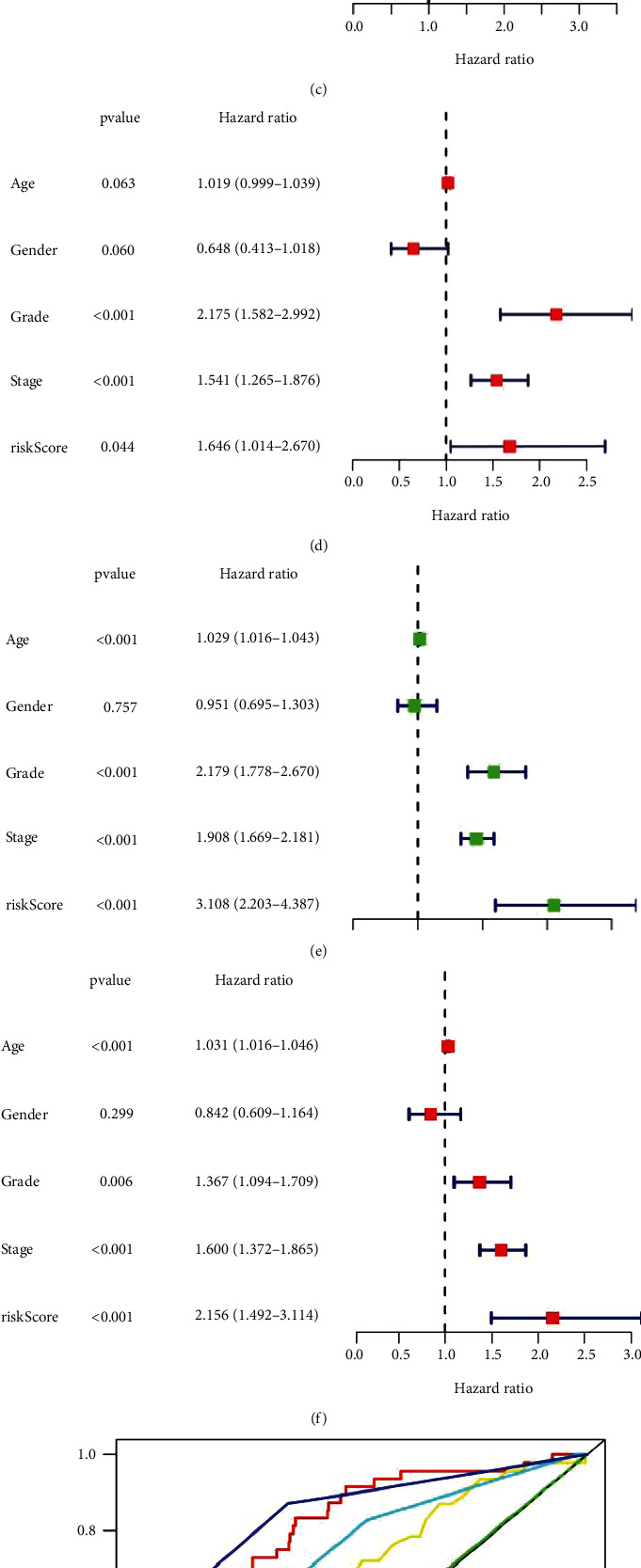
Validation of risk signature power and independent prognostic factors. (a), (c), and (e) Uni-Cox and (b), (d), and (f) multi-Cox analysis of clinicopathological factors and the risk scores in the training set, testing set, and the whole set. (g) The area under the curve (AUC) and the receiver operating characteristic (ROC) curves of different factors. (h) The AUC under the 1-, 3-, and 5-year ROC curves of risk scores.

The predictive applicability of our signature as an independent feature for ccRCC prognosis was evaluated by uni-Cox regression analysis, which revealed that ccRCC patients' OS was substantially related to their stage and risk score in the training (Figure 5(a)) and test set (Figure 5(c)), as well as the overall data set (Figure 5(e)). Multi-Cox regression analysis suggested that disease stage and risk score are all capable to independently predict OS in three data sets (Figures 5(b), 5(d), and 5(f)). Then, in the complete collection, we validated the signature's sensitivity and specificity via area under the curve (AUC) analysis. Our signature's AUC was 0.793, which was the second best predictor of patient survival after disease stage (Figure 5(g)). The 1-, 3-, and 5-year survival AUCs were 0.793, 0.716, and 0.719, respectively, all of which indicate reliable predictions (Figure 5(h)). These findings suggested that our signature is a biomarker for ccRCC prognosis.

### 3.5. The Prognostic Nomogram

**Figure 6:**
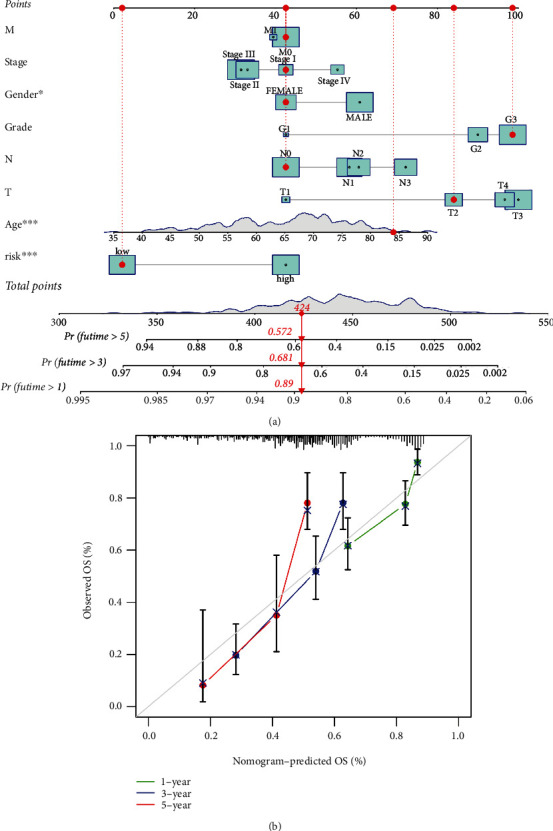
The nomogram of prognostic model. (a) A nomogram combining age, gender, pathological grade, stage, tumor stage, node stage, metastasis stage, and risk score that predicts the 1-, 3-, and 5-year prognoses of clear cell renal cell carcinoma patients. (b) The 1-, 3-, and 5-year calibration curves for the nomogram.

A nomogram with clinicopathological characteristics and risk scores taken into account was created to predict the prognosis of ccRCC patients after 1, 3, and 5 years (Figure 6(a)). All the calibration curves revealed a good match between the anticipated and actual survival rates (Figure 6(b)).

### 3.6. The Cuproptosis-Related lncRNAs signature's TIME

**Figure 7:**
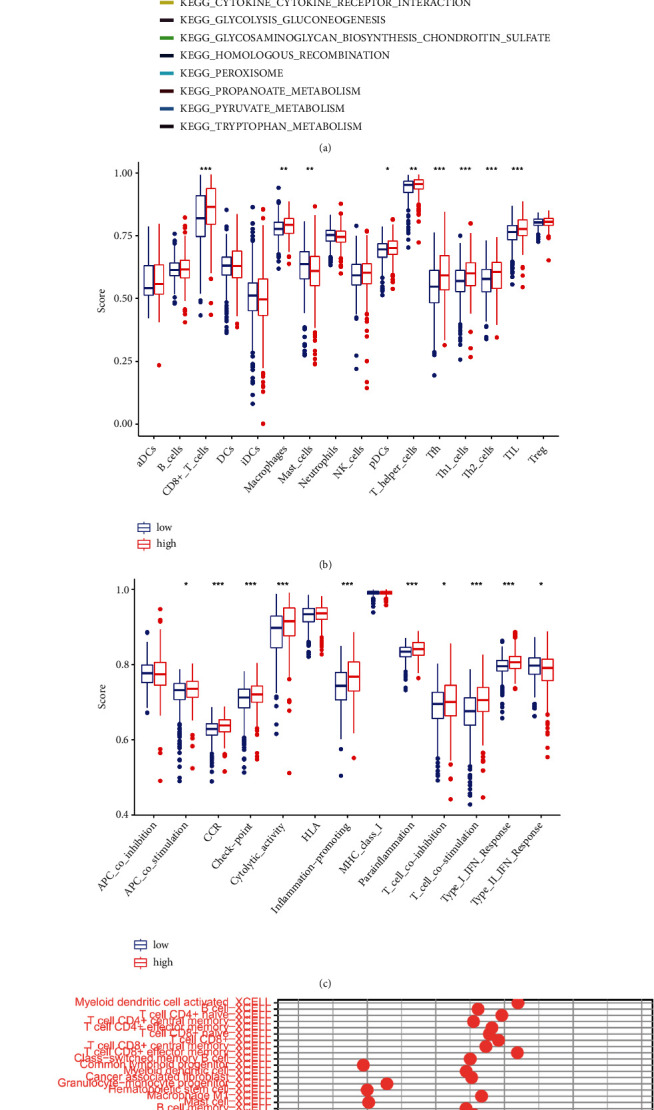
The correlations between risk signatures and immune infiltration. (a) Top 10 most enriched pathways in both risk groups as analyzed by Gene Set Enrichment Analysis (GSEA). (b) Infiltration of immune cells in the two groups as obtained by single-sample GSEA. (c) The immune functions of the two groups as obtained by single-sample GSEA. (d) Bubble plot of the correlations between risk scores and immune cells under different platforms. ∗*p* < 0.05, ∗∗*p* < 0.01, and ∗∗∗*p* < 0.001.

The GSEA results showed a significant enrichment in the TCA cycle, pyruvate metabolism, butanoate metabolism, citrate cycle, glycolysis gluconeogenesis, peroxisome, propanoate metabolism, and tryptophan metabolism of the low-risk group. On the other hand, glycosaminoglycan biosynthesis chondroitin sulfate, cytokine–cytokine receptor interactions (CCRIs), and homologous recombination are biological processes highly enriched in the high-risk group (Figure 7(a)). Besides, ssGSEA results found that tumor-infiltrating lymphocytes, macrophages, plasmacytoid dendritic cells (pDCs), T helper cells, T follicular helper (Tfh) cells, Th1, Th2, and CD8^+^ T cells were markedly more prevalent in the high-risk group (Figure 7(b)). Moreover, the high-risk group also demonstrated more advanced levels of several immune functions than the low-risk group (Figure 7(c)).

**Figure 8:**
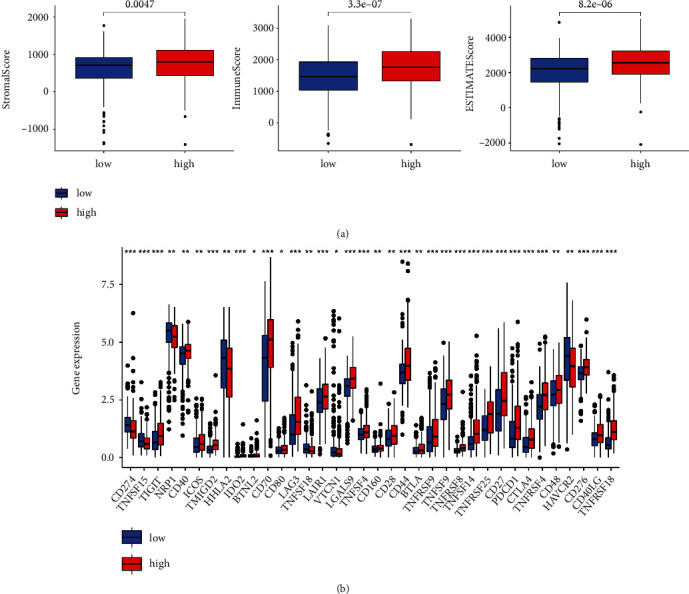
The tumor immune microenvironment and immune checkpoints between the two groups. (a) Boxplots of StromalScore, ImmuneScore, and ESTIMATE Score between the two groups. (b) Immune checkpoint expressions between the two groups. ∗*p* < 0.05, ∗∗*p* < 0.01, and ∗∗∗*p* < 0.001.

**Figure 9:**
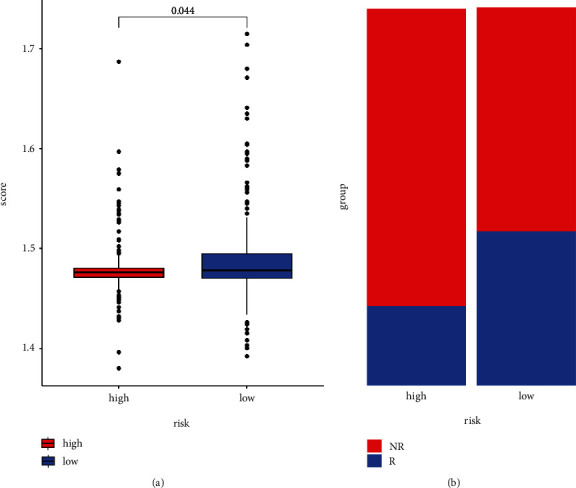
Response to immunotherapy in high- and low-risk groups. (a) Scores for immune responses in two groups. (b) Proportion of immune responses in two groups.

Additionally, more immune cells were found positively correlated with the risk score (Figure 7(d); Supplementary Table S2), and high-risk patients also revealed greater StromalScore, ImmuneScore, and ESTIMATE than the low-risk ones (Figure 8(a)). Immune checkpoint (ICP) is an immunological modulator that has been linked to cancer therapy as an indicator of immune response. As a result, we looked at the expression of ICP-related modulatory genes in the TCGA database (Figure 8(b)). 36 ICP-related genes were found to express differently between the two groups, and several immune checkpoint inhibitors showed high expression in the high-risk group. These findings all showed that the more activation of immune system was found in high-risk group. Furthermore, ImmuCellAI's predictions showed how patients respond to ICB. A higher immune score indicated a better response to ICB, so the patients in the low-risk group responded better to ICB than those in the high-risk group (Figure 9(a)). In addition, in the low-risk group, a higher proportion of patients responded to ICB, but a higher proportion of patients did not respond to ICB in the high-risk group.

## 4. Discussion

Considering the multifactorial proteogenomic and genetic features, ccRCC is a highly heterogeneous RCC subtype, leading to worse prognoses [19]. Previous studies reported lncRNAs in the oncogenesis of ccRCC importantly, which could help the treatment of ccRCC as effective and potential molecular targets [20]. lncRNA has been shown to regulate cell processes like metabolism, inflammation, immune response, and autophagy [21–24]. As of today, studies have revealed that some lncRNAs could participate in ccRCC progression by regulating the occurrence [25]. Research studies have been established on the relationship between ferroptosis and ccRCC with some achievements. Until now, the cuproptosis-related lncRNAs signature has not been found in other known studies. Therefore, we proposed a cuproptosis-related lncRNAs signature that may have the potential for prognosis and prediction. Our findings might contribute to elucidating therapeutic targets and potential genomic biomarkers in the cuproptosis-related signaling pathways.

First, we used multivariate, LASSO, and univariate Cox regression analyses to identify 5 cuproptosis-related lncRNAs (FOXD2-AS1, LINC00460, AC091212.1, AC007365.1, and AC026401.3) in the end, which were included in the predictive signature. FOXD2-AS1 has been reported to modulate PI3K/AKT and HMGA2 downstream signaling by sponging miR-185-5p, thereby promoting tumorigenesis and tumor progression in glioma [26]. Another study found that the expression of LINC00460 is independently associated with the OS in terms of the lymph node metastasis and Tumor Node Metastasis (TNM) stages [27]. Besides, this lncRNA has been proved to participate in ccRCC pathogenesis and development through the ferroptosis signal passway, which suggests the significance of prognosis potential [28]. We also applied three other lncRNAs (AC091212.1, AC007365.1, and AC026401.3), which have not been previously reported in ccRCC, and further studies are needed to elucidate their mechanisms in oncogenesis. Furthermore, we investigated how TIME, immune checkpoint inhibitors, and immune infiltrating cells affect ccRCC prognosis.

Our GSEA analysis showed that the chondroitin sulfate biosynthesis pathway, CCRIs, and homologous recombination pathway were highly enriched in high-risk patients. The TCA cycle and other related pathways, such as the pyruvate metabolism pathway, gluconeogenesis pathway, and the tryptophan metabolism pathway, were primarily enriched in low-risk patients [29]. AURKB can promote the ccRCC development via CCRI as one of the signaling pathways [30]. CXCL5 also has a higher expression in ccRCC [31], which role in metastasis, tumor development, and angiogenesis [32], has led to its designation as a key biomarker and supplementary antiangiogenic treatment target [33]. In venous tumor thrombus cases, there was an increased incidence of homologous recombination repair genes in ccRCC patients [34]. These results indicated that pathways that enriched in high-risk patients are correlated with poorer prognosis.

Recently, a study showed cuproptosis in human cells mainly by affecting lipoylated TCA cycle proteins [5]. FDX1, one of the related genes, encodes a reductase to activate production of Cu^1+^, which has higher toxicity than Cu^2+^, playing an important role in the cuproptosis signal pathway [35]. Depleting mitochondrial copper can switch metabolism from respiration to glycolysis to lower energy output, which has been shown to be beneficial against cancers depending on oxidative phosphorylation [36]. In previous studies, the expression level of copper transporters 1 and 2 has been related to the prognosis of ccRCC, probably by hypoxia-inducible factor imbalance [37, 38]. Consistent with our results, overexpression of cuproptosis-related genes may increase necrosis in tumor cells, leading to a better ccRCC prognosis.

Subsequent ssGSEA results showed that Tfh, Th1, Th2, neutrophils, macrophages, Tregs, and mast cells revealed greater scores in the high-risk group. Besides, this group demonstrated greater expressions of inflammation-promoting and parainflammation factors as well as more intense type I Interferon (IFN) response, indicating a decreased autoimmunity function and a poor prognosis. If tumor-associated macrophages reveal a high infiltration, a poor OS in metastatic ccRCC could be expected [39]. In ccRCC patients, tumor-infiltrating mast cells secret IL-10 and TGF-*β* to decrease anti-tumor immunity, leading to a worse prognosis [40].

RCC lesions are highly infiltrated by immune cells, especially CXCL13^+^CD8^+^ T cells, which enclose a higher level of immune checkpoints. In ccRCC patients, higher infiltration of CXCL13^+^CD8^+^ T cells weakens the total immunological function, and intratumoral CXCL13^+^CD8^+^ T cell infiltration can lead to a worse clinical prognosis [41]. The high CD8^+^ T cell infiltration found in this study was consistent with the previous findings [42].

Besides, the cuproptosis-related lncRNAs signature proposed in this study was correlated with CD44, CD70, CTLA4, and other ICIs, all of which were expressed at a greater amount in the high-risk group [43]. Immunotherapy has become promising as a treatment strategy for ccRCC currently [44]. With the advent of molecular and genomic research, ICB immunotherapy, as a novel strategy, has been proved in the improvement of ccRCC patients [45]. According to our results, compared with the low-risk group, patients in the high-risk group with elevated immune checkpoint-related gene expression and CD8^+^ T cell infiltration were less sensitive to ICB therapy, which is consistent with previous results that both the CD8^+^ T cells infiltration and PD-1 expression [42, 46] cannot define ccRCC prognosis and guide ccRCC therapies, our prognostic signature would help fill this blank. Our results may contribute to the development of personalized immunotherapy for patients with ccRCC. However, deeper and wider elucidations are needed with regard to the functions of cuproptosis-related genes in ccRCC. Overall, these findings suggested that the cuproptosis-related lncRNAs signature may predict the level of immune checkpoints expression and guide the ICB immunotherapy process.

However, there were certain limitations to the study. First, independent external validation datasets were lacked, and clinical sample sizes were limited, which could lead to untrustworthy results. Second, complicating factors, such as comorbidities, might influence signature accuracy and robustness. Finally, our research was limited to theoretical research based on bioinformatics and statistical analysis. Future studies would require biochemical studies and animal experiments to corroborate the findings.

## 5. Conclusion

We proposed a specific cuproptosis-associated lncRNAs signature for the outcome of ccRCC patients, which could independently predict the prognosis. Our signature might help explore the mechanism of cuproptosis-related lncRNAs in ccRCC and offer ccRCC patients potential therapeutic targets.

## Figures and Tables

**Table 1:** Characteristics of clear cell renal cell carcinoma patients.

Characteristics	Entire cohort (513), *N* (%)	Training cohort (257), *N* (%)	Validation cohort (256), *N* (%)	*p*-Value
Age (years)				
≤60	262 (51.1)	134 (52.1)	128 (50)	0.628
>60	251 (48.9)	123 (47.9)	128 (50)	
Gender				
Female	176 (34.3)	89 (34.6)	87 (34.0)	0.878
Male	337 (65.7)	168 (65.4)	169 (66.0)	
Stage				
I	255 (49.7)	121 (47.1)	134 (52.3)	0.700
II	56 (10.9)	28 (10.9)	28 (10.9)	
III	117 (22.8)	62 (24.1)	55 (21.5)	
IV	82 (16.0)	45 (17.5)	37 (14.5)	
Unknown	3 (0.6)	1 (0.4)	2 (0.8)	
Stage T				
T1	261 (50.9)	125 (48.6)	136 (53.1)	0.401
T2	68 (13.3)	35 (13.6)	33 (12.9)	
T3	173 (33.7)	89 (34.6)	84 (32.8)	
T4	11 (2.1)	8 (3.1)	3 (1.2)	
Stage N				
N0	229 (44.6)	114 (44.4)	115 (44.9)	0.566
N1	16 (3.1)	6 (2.3)	10 (3.9)	
Unknown	268 (52.2)	137 (53.3)	131 (51.2)	
Stage M				0.430
M0	407 (79.3)	198 (77.0)	209 (81.6)	
M1	78 (15.2)	43 (16.7)	35 (13.7)	
Unknown	28 (5.5)	16 (6.2)	12 (4.7)	
Grade				0.290
G1	12 (2.3)	4 (1.6)	8 (3.1)	
G2	219 (42.7)	108 (42.0)	111 (43.4)	
G3	201 (39.2)	106 (41.2)	95 (37.1)	
G4	78 (15.2)	39 (15.2)	39 (15.2)	
Unknown	3 (0.6)	0 (0.0)	3 (1.2)	
Aeoadjuvant treatment				0.464
Yes	17 (3.3)	10 (3.9)	7 (2.7)	
No	496 (96.7)	247 (96.1)	249 (97.3)	
Alive status				0.901
Alive	344 (67.1)	173 (67.3)	171 (66.8)	
Died	169 (32.9)	84 (32.7)	85 (33.2)	

## Data Availability

Our study made use of public databases and this data could be found on https://portal.gdc.cancer.gov/.

## Abbreviations

ccRCC: Clear cell renal cell carcinoma
TCA: Tricarboxylic acid
lncRNA: Long non-coding RNAs
TCGA: The Cancer Genome Atlas
LASSO: Least Absolute Shrinkage and Selection Operator
ROC: Receiver operating characteristic
AUC: Area under the curve
GSEA: Gene Set Enrichment Analysis
ICB: Immune-checkpoint blockade.

## Supplementary Materials

Supplementary Materials Supplementary Figure S1: The diagram of our study flow.

Supplementary Materials Supplementary Table S1: 5 cuproptosis-related lncRNAs (FOXD2-AS1, LINC00460, AC091212.1, AC007365.1, and AC026401.3) and its regression coefficients derived from the results of multi-Cox regression analysis.

Supplementary Materials Supplementary Table S2: Correlation between risk score and immune cell infiltration data generated by 7 different platforms.
