# Supplementary figures and images for: A Cuproptosis-Related lncRNAs Signature Could Accurately Predict Prognosis in Patients with Clear Cell Renal Cell Carcinoma
Source: Anal Cell Pathol (Amst). 2022 Dec 22;2022:4673514. doi: 10.1155/2022/4673514 (PMC9800904; doi:10.1155/2022/4673514)

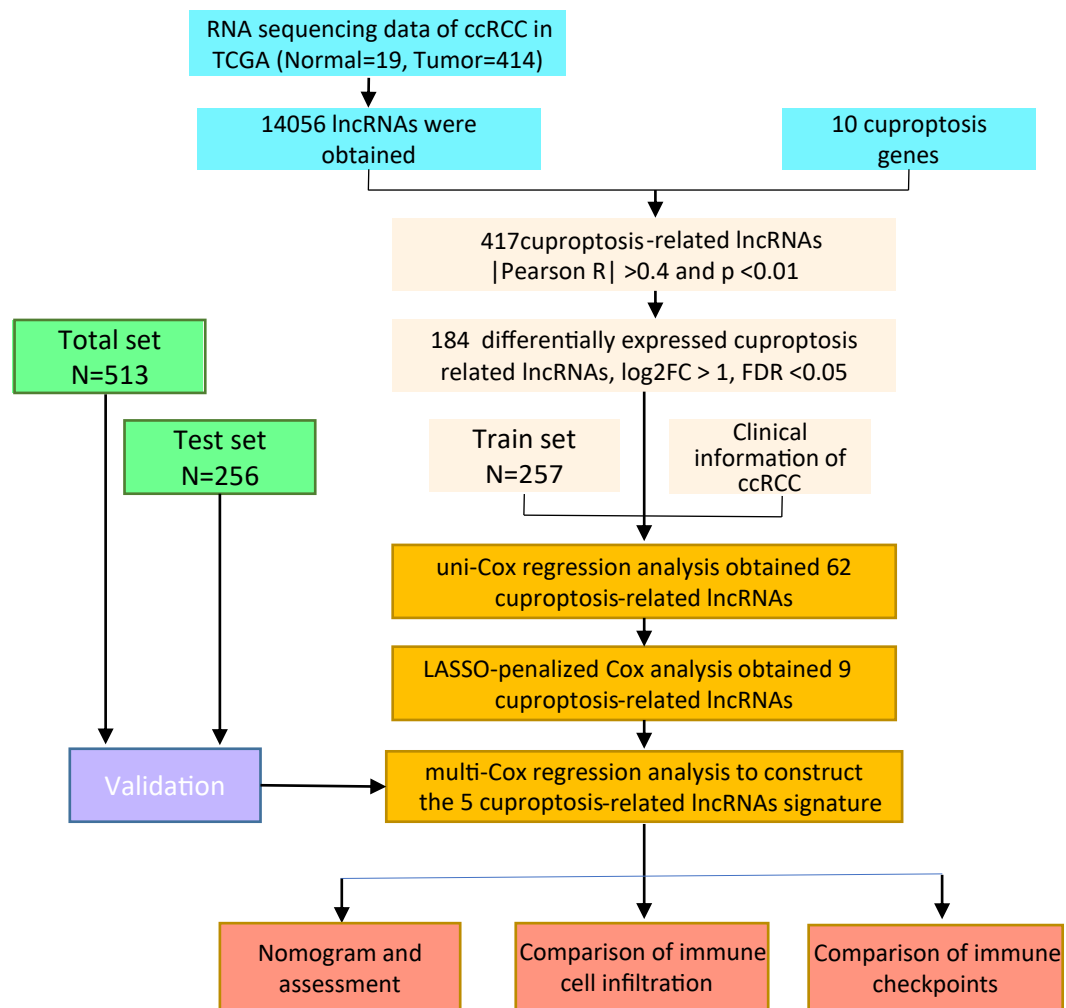

Supplement: Supplementary Materials — Supplementary Figure S1: The diagram of our study flow. [file 4673514.f1.pdf]
